# Generation and characterization of a mouse model of conditional *Chd4* knockout in the endometrial epithelium

**DOI:** 10.1371/journal.pone.0326723

**Published:** 2025-12-19

**Authors:** Shannon K. Harkins, Hilary J. Skalski, Abigail Z. Bennett, Laura A. Pavliscak, Nakati C. Sany, Amelia R. Arendt, Lauren Wood, Genna E. Moldovan, Ronald L. Chandler

**Affiliations:** Department of Obstetrics, Gynecology, and Reproductive Biology, College of Human Medicine, Michigan State University, Grand Rapids, Michigan, United States of America; Universite du Quebec a Trois-Rivieres, CANADA

## Abstract

Chromatin remodeling plays an integral part in endometrial homeostasis through its roles in the maintenance of cell identity, epithelial integrity, and prevention of endometrial disease. Chromodomain-helicase-DNA-binding protein 4 (CHD4) is a chromatin remodeling protein and member of the NuRD complex, which predominantly represses transcription. *CHD4* is mutated in endometrial carcinoma, with most mutations resulting in loss of function. *CHD4* has been identified as a tumor suppressor and regulator of stemness in human endometrial carcinoma cell lines, but little is known about the tissue-specific roles of *CHD4* in the endometrial epithelia *in vivo.* We generated a conditional *Chd4* floxed allele and combined it with *BAC-Sprr2f-Cre* to drive CHD4 loss in the endometrial epithelium. Consistent with previous reports, *BAC-Sprr2f-Cre* shows variegated expression within the endometrial epithelium and lacks expression in the oviducts, ovaries, and kidneys. Loss of CHD4 was confirmed by immunohistochemistry, and the percentage of endometrial epithelial cells with and without CHD4 was quantified. Compared to the glandular epithelium, the extent of CHD4 loss was higher in the luminal epithelium and unaffected by age. Mice with conditional knockout of *Chd4* had normal endometrial histology. A six-month breeding trial was performed to assess the functional effects of endometrial epithelial CHD4 loss on fertility and found no difference in litter size, average litter size per dam, or pup weight between genotypes. These findings demonstrate that *Chd4* conditional knockout using *BAC-Sprr2f-Cre* is not sufficient to alter the structure and function of the endometrial epithelium or drive tumorigenesis. As *CHD4* is frequently co-mutated with other cancer driver genes such as *TP53*, *PIK3CA*, and *PTEN*, future mouse modeling efforts emulating patient mutational profiles might provide insight into the role of *CHD4* in endometrial carcinoma.

## Introduction

The endometrium is a dynamic, hormone-responsive tissue that forms the inner uterine lining and consists of luminal epithelium, glandular epithelium, and stroma. The primary physiological function of the endometrium is to prepare for and maintain pregnancy [1]. In response to ovarian steroid hormones, estrogen and progesterone, the endometrium undergoes cyclic proliferation and breakdown each menstrual cycle. Endometrial function is dependent upon the ability of endometrial tissue to respond to hormonal signals through coordinated transcriptional programs, a process mediated in part by chromatin remodeling [2–4].

Chromatin remodeling genes play an integral role in the maintenance of endometrial homeostasis [2–4], and their disruption through mutations or alterations is associated with endometrial pathologies, including endometriosis [5], endometrial hyperplasia [6], and 66% of endometrial carcinomas [7]. Chromatin remodeling proteins function by using energy from ATP hydrolysis to alter nucleosome structure, chromatin accessibility, and ultimately, gene transcription [8,9]. We previously showed that SWI/SNF chromatin remodeling proteins, ARID1A and BRG1, regulate endometrial epithelial identity and integrity, with the loss of SWI/SNF leading to an epithelial-to-mesenchymal transition (EMT) phenotype in mice [10,11].

Chromodomain-helicase-DNA-binding protein 4 (CHD4) is an ATP-dependent chromatin remodeling protein and integral subunit of the nucleosome remodeling and deacetylase (NuRD) complex, which regulates transcriptional repression, DNA damage response, and cell cycle progression [12–17]. *CHD4* is the predominant chromatin remodeling gene mutated or altered in an aggressive form of endometrial carcinoma known as uterine serous carcinoma. Approximately 17% of cases harbor *CHD4* mutations [18,19], with the majority of mutations resulting in loss of CHD4 function [20,21]. CHD4 has been shown to have tumor suppressive properties in human endometrial carcinoma cell lines, with CHD4 loss leading to increased invasion and stemness *in vitro* [20]. *CHD4* has been associated with the acquisition of a metastatic phenotype in several cancer types, including ovarian, colorectal, papillary thyroid, and breast cancers [22–25]. However, little is known about the tissue-specific functions of CHD4 within the normal endometrium *in vivo* or the extent to which CHD4 loss may contribute to endometrial disease. To this end, we generated a conditional *Chd4* floxed allele, which, when combined with *BAC-Sprr2f-Cre,* targets *Chd4* knockout to the endometrial epithelium [26], the proposed cell of origin for endometrial carcinoma [27]. We created the first endometrial epithelial-specific conditional knockout of *Chd4* and characterized the structural and functional consequences of CHD4 loss.

## Materials and methods

### Ethics statement

All mouse experiments were performed under protocol #: 202500068, approved by the Michigan State University (MSU) Institutional Animal Care and Use Committee and in compliance with the National Institutes of Health’s Guide for the Care and Use of Laboratory Animals. Mice were housed at the MSU Grand Rapids Research Center Vivarium on a standard 12-hour light-dark cycle with ad libitum food and water. Mice were humanely euthanized by CO_2_ inhalation delivered by an automated Euthanex System.

### Mice

All mice were maintained on a C57BL/6 background. The C57BL/6N-*Chd4*^*tm1a(EUCOMM)Wtsi*^*/BayMmucd* (knockout-first, conditional *Chd4* floxed-neo allele, *Chd4*^*fl-neo*^) allele was obtained from Mutant Mouse Resource and Research Centers at the University of California Davis (MMRRC #037690-UCD) [28]. The *BAC-Sprr2f-Cre* (strain #: 037052) [26], R26-Flp knock-in (*R26*^*Fki*^; strain #: 016226) [29], and R26-mT/mG (*R26*^*mT/mG*^; strain #: 007676) [30] alleles were purchased from The Jackson Laboratory (Bar Harbor, ME). Inheritance of the *BAC-Sprr2f-Cre*, the *R26*^*Fki*^, and the *R26*^*mT/mG*^ alleles was confirmed by PCR using published methods [30–32]. ; *Ep400^fl^^^/^fl^ or Ep400*^fl/+^ floxed (fl/fl or fl/+) mice were used as control mice unless otherwise specified.

### Generating the conditional *Chd4* floxed allele

The *Chd4*^*fl-neo*^ allele configuration was generated through a targeted breeding scheme that induced sequential recombination by FLP1 and Cre recombinase. The presence and configuration of the *Chd4*^*fl-neo*^ allele were confirmed by PCR in accordance with MMRC-UCD genotyping protocol guidelines [33]. Table 1 summarizes the primer sequences, primer combinations, and PCR product sizes for each allele configuration of the *Chd4*^*fl-neo*^ allele [33]. Reactions highlighted in grey were not performed or included. PCR reaction conditions consisted of initial denaturation at 94°C for 2 minutes, followed by 34 cycles at 94°C for 15 seconds, 58°C for 30 seconds, and 72°C for 1 minute, and ended with extension at 72°C for 5 minutes. Agarose gels were imaged using a ChemiDoc XRS+ System (Bio-Rad Laboratories).

**Table 1:** PCR Genotyping of the conditional *Chd4* floxed allele configurations.

Primer Name	Nucleotide Sequence (5’ – 3’)	Primer Combination	Product size (bp)	Genotype	Allele configuration	Allele
1. 37690-lacF	GCTACCATTACCAGTTGGTCTGGTGTC	3 & 5	320	floxed	tm1a, tm1c	*Chd4*^*fl-neo*^, *Chd4*^*fl-only*^
2. 37690-neoF	GGGATCTCATGCTGGAGTTCTTCG	2 & 4	536	PreCre, PreFlp	tm1a	*Chd4* ^*fl-neo*^
3. 37690-loxF	GAGATGGCGCAACGCAATTAATG	1 & 5	534	PostCre	tm1b	*Chd4* ^*Δ*^
4. 37690-TTR	ACCTGCAGCCTACTGCCATGG	6 & 4	291	Wild-type	–	*Chd4* ^*+*^
5. 37690-R	GAGATGGCTCAGTGGGTAAGAGTACC	6 & 4	413	PostFlp	tm1c	*Chd4* ^*fl-only*^
6. 37690-F	GCAGTTCTGAGTGTAAGGTCAGTCTGG	6 & 5	460	PostFlp & PostCre	tm1d	*Chd4* ^*Δ*^

### Estrous cycle staging

Vaginal lavage cytology was used to determine the murine estrous cycle stage as described [34]. Twelve-week-old pilot mice were not estrous cycle staged. Mice in the 26-week-old cohort were sacrificed in the diestrus phase of the estrous cycle.

### X-gal staining

X-gal staining solution and wash buffer were prepared as described, except that the staining solution contained 20 mM Tris-HCl (pH 7.5) [35]. All wash steps were repeated three times using wash buffer. Isolated organs were fixed in 4% paraformaldehyde (PFA) in phosphate-buffered saline (PBS) overnight at 4°C, washed, stained with X-gal staining solution overnight, washed, and placed in 4% PFA overnight for post-staining fixation. Samples were then washed and cleared through a graded glycerol series (50%, 70%, 90%, 100%) with consecutive overnight incubations.

### Whole-mount tissue imaging

Whole-mount tissue samples, including X-gal-stained tissues and *R26*^*mT/mG*^ mice, were imaged using a Nikon SMZ18 Stereo Microscope with either a 0.5x or 1x SHR Plan Apo objective, illuminated by a KL 1600 LED light source (SHOTT) for brightfield imaging or an X-Cite Series 120PC Q (EXFO) for fluorescence imaging. Images were captured using a Nikon DS-U3 camera and the NIS-Elements Software (Nikon Instruments Inc., Tokyo, Japan).

### Tissue processing and histology

Upon necropsy, one uterine horn was fixed in 10% neutral buffered formalin in PBS for 72 hours. Samples were then washed twice with PBS and transferred to 70% ethanol. Samples were submitted to the Van Andel Research Institute (VAI) Pathology & Biorepository Core for paraffin embedding, sectioning, and hematoxylin and eosin (H&E) staining. The other uterine horn was fixed in 4% paraformaldehyde (PFA) for at least 72 hours and stored at 4°C. Each uterine horn was washed twice with PBS, subjected to a sucrose gradient (24 hours in 15% sucrose in PBS followed by 24 hours in 30% sucrose in PBS), embedded in O.C.T. Compound (Fisher Health Care), and cryosectioned as described [11].

### Immunofluorescence (IF)

IF was performed as described [11]. Slides were blocked using donkey blocking solution: 5% normal donkey serum (Jackson Immunoresearch Laboratories # 017-000-121), 1% IgG-free bovine serum albumin (Jackson Immunoresearch Laboratories # 001-000-161), and 0.05% Tween 20 in PBS. Cryosections were co-stained with chicken anti-GFP (Abcam #ab13970, 1:500) and rat anti-KRT8 (DSHB #TROMA1, 1:100). The following secondary antibodies were purchased from Jackson Immunoresearch Laboratories and used at a 1:250 dilution: donkey anti-chicken AF 488 (JAX #703-545-155) and donkey anti-rat AF 647 (JAX #712-605-153). Autofluorescence reduction was performed using the TrueVIEW Auto-fluorescence Quenching Kit (Vector Laboratories #SP-8500). Slides were mounted and stained with DAPI using ProLong Gold Antifade Reagent with DAPI (Invitrogen). Slides were imaged using the Nikon C2 Plus Confocal microscope configured on a Nikon Eclipse Ti inverted microscope using a 40x Plan Fluor oil objective (NA 1.30). DAPI, GFP, and KRT8 were excited using 405, 488, and 637 nm laser lines, respectively. Emission was collected by PMT detectors at 500–550 nm for GFP and 660–1000 nm for AF647. DAPI emission was collected using the closest available detector window.

### Immunohistochemistry

Indirect immunohistochemistry (IHC) was performed as described [11,34]. Antigen retrieval was performed using 10mM sodium citrate buffer Primary antibodies were incubated overnight at the following dilutions: 1:200 anti-CHD4 (Cell Signaling Technology, CST #12011), 1:250 anti-Cleaved caspase 3 (CC3; CST # 9579), 1:400 anti-Ki-67 (CST #12202), 1:100 anti-Keratin 8 (KRT8; Developmental Studies Hybridoma Bank #TROMA-I), 1:400 anti-E-Cadherin (CST# 3195), and 1:400 anti-GFP (CST#2956). Stained slides were digitally scanned at 20x magnification and imported into Aperio eSlide Manager by the VAI Pathology & Biorepository Core. Histology images were acquired using Aperio ImageScope 12.4.6 software (Leica Biosystems).

### IHC quantification

For each mouse, a representative anti-CHD4 and anti-Ki-67 IHC image was taken at 20x magnification. Aperio eSlide Manager was selected for quantification of endometrial epithelial CHD4 and Ki-67 expression. The multipoint tool in Fiji/Image J was used to manually count DAB-positive and DAB-negative endometrial epithelial cells for anti-CHD4 or anti-Ki-67 IHC. Counts for the luminal and glandular epithelia were enumerated separately and were combined to calculate the counts for total epithelia. The percentage of cells expressing CHD4 or Ki-67 was calculated by dividing the number of DAB-positive cells by the total number of cells (DAB-positive + DAB-negative cells) and multiplying by 100% for the luminal, glandular, and total epithelia.

### Statistical analysis

All statistical analyses were performed using GraphPad Prism 10. Scatter plots show the mean and standard deviation for each dataset. An unpaired, two-tailed t-test was used to assess differences between genotypes unless otherwise specified. Welch’s Correction was used for samples with unequal variance. Significance thresholds: ns = not significant; p < 0.05 = *, p < 0.01 = **, p < 0.001 = ***, p < 0.0001 = ****.

## Results

### Creating the conditional *Chd4* floxed-only allele

**Fig 1:**
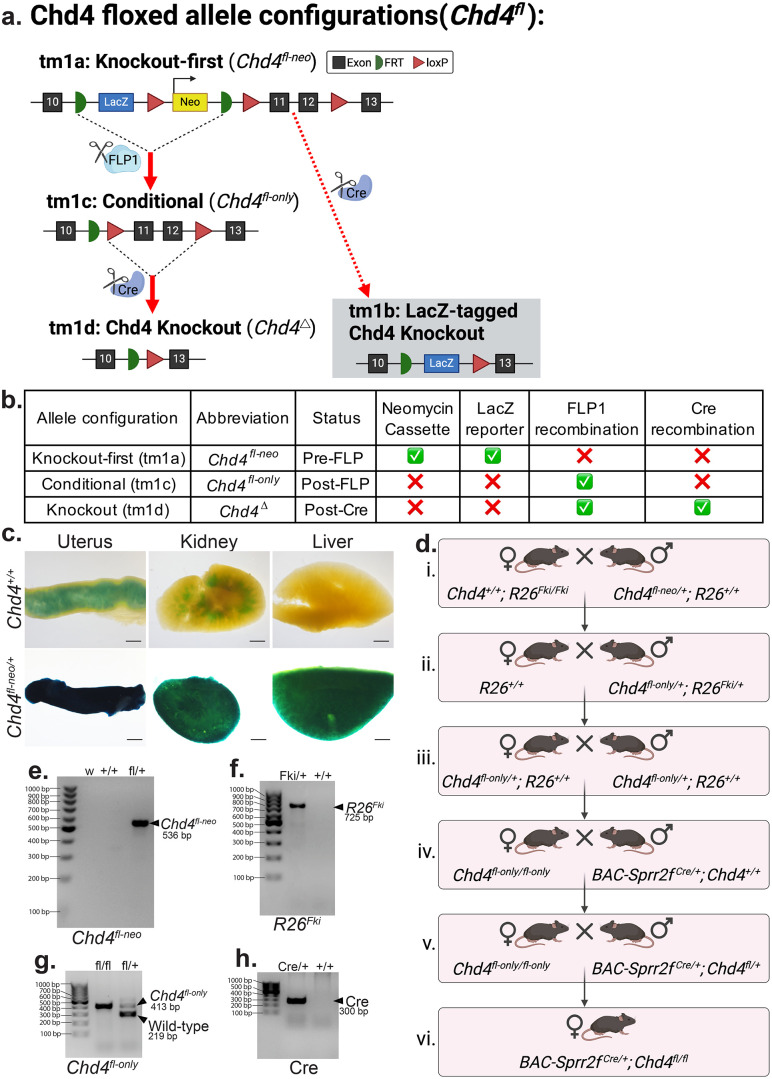
Generating the *Chd4* conditional knockout mouse. **a.** Schematic of the knockout-first, conditional *Chd4* floxed allele. The targeted mutation 1a (tm1a) allele configuration contains flippase recognition targets (FRT) flanking a *lacZ* reporter and a neomycin resistance cassette. LoxP sites flank the neomycin cassette and critical exons 11 and 12. The tm1b allele configuration (gray box) is produced when Cre recombinase is introduced prior to FLP1 recombination (not generated in this study). The tm1c allele configuration is produced following FLP1 recombination. The tm1d allele configuration is only produced in cells expressing Cre recombinase. The use of *BAC-Sprr2f-Cre* drives conditional knockout of the *Chd4* floxed allele in the endometrial epithelium. **b.** Tabular summary of the tm1a, tm1c, and tm1d allele configurations for the *Chd4*^*fl-neo*^ allele. **c.** X-gal staining was used to assess the functionality of the *lacZ* reporter gene in mice within the *Chd4*^*fl-neo*^ allele in *Chd4*^*fl-neo*/^^+^ and control (*Chd4*^+^^*/*^^+^ ) mice. The *lacZ* reporter gene allows cells with the *Chd4*^*fl-neo*^ allele to produce β-galactosidase, which hydrolyses X-gal and produces a blue/green color. The control uterus and the kidney had some background staining, which could result from endogenous β-galactosidase activity known to occur in these two organs [37]. Scale bar = 1000 µm. **d.** A selective breeding scheme was used to refine the *Chd4*^*fl-neo*^ allele to the *Chd4*^*fl*^ allele to ultimately lead to endometrial epithelial *Chd4* knockout. **i.** A female *R26*^*Fki*^ homozygote was crossed with a *Chd4*^*fl-neo/*^+ sire. **ii.** FLP1 recombination occurred in offspring that inherit both the *R26*^*Fki*^ and *Chd4*^*fl-neo*^ alleles, creating the *Chd4*^*fl*^ allele. The *R26*^*Fki*^ allele was no longer needed and was bred out by crossing *Chd4*^*fl/*^^+^ *; R26*^*Fki*^ with a wild-type C57BL/6 mouse. **iii.** The resultant *Chd4*^*fl/*^^+^ offspring were crossed to create female *Chd4*^*fl-only*^ homozygotes. **iv.** A *BAC-Sprr2f-Cre* ^0/+^*Chd4*^+ ^*/*^+^ sire is crossed with a *Chd4*^*fl/fll*^ dam. **v.** The resultant *BAC-Sprr2f-Cre*^0/+^; *Chd4*^*fl/*^^+^ sires were then crossed with *Chd4*^*fl/fl*^ dams. **vi.** The resultant *BAC-Sprr2f* -*Cre^0^^*/*^^+^; Chd4*^*fl/fl*^ female offspring had endometrial epithelial *Chd4* knockout. **e.**-**h.** Representative PCR genotyping gels to confirm: the presence of the *Chd4*^*fl-neo*^ allele at 536 bp (**e**), the *R26*^*Fki*^ at 725 bp (**f**), the *Chd4*^*fl*^ allele at 413 bp and the wild-type allele at 219 bp (**g**), and the *Cre* allele *for BAC-Sprr2f-Cre* mice at 300 bp (**h**).

The knockout-first, conditional *Chd4* floxed-neo allele (*Chd4*^*tm1a(EUCOMM)Wtsi*^*/Chd4*^*fl-neo*^) was obtained from the Mutant Mouse Resource and Research Center in the targeted mutation 1a (tm1a) configuration. The *Chd4*^*fl-neo*^ allele contains a lacZ reporter and neomycin selection cassette flanked by flippase recognition target (FRT) sites and loxP sites flanking critical exons 11 and 12 (Fig 1a-b)(#037690-UCD) [28]. The presence of the *Chd4*^*fl-neo*^ allele was confirmed by X-gal staining. The LacZ reporter present in the *Chd4*^*fl-neo*^ allele hydrolyzes X-gal substrate, whose product produces a blue precipitate upon further oxidation [36]. This color change was apparent in the uterus, kidney, and liver from the mouse containing the *Chd4*^*fl-neo*^ allele (Fig 1c).

A targeted breeding scheme was performed to achieve conditional knockout of *Chd4* exclusively in the endometrial epithelium driven by *BAC-Sprr2f-Cre* (JAX: #037052) (Fig 1d) [26]. The lacZ reporter and neomycin selection cassette present in the *Chd4*^*fl-neo*^ allele are not needed *in vivo* and were removed to prevent off-target activity [38]. The first step toward FLP1 recombination was crossing a sire heterozygous for the *Chd4*^*fl-neo*^ allele with a dam homozygous for the R26-Flp knock-in allele (*R26*^*Fki*^; JAX #016226) (Fig 1d, i) [29]. FLP1 recombination occurred in the resultant offspring that inherit both the *R26*^*Fki*^ *and Chd4*^*fl-neo*^ alleles, producing the *Chd4*^*fl*^ allele (Fig 1d, ii). To prevent future FLP1 recombination events, *Chd4*^*fl-only*^*; R26*^*Fki*^ offspring were crossed with a wild-type C57BL/6 mouse to remove the *R26*^*Fki*^ allele (Fig 1d, ii). The resultant offspring that were heterozygous for the *Chd4*^*fl*^ allele were then crossed to produce female *Chd4*^*fl-*^ homozygotes (Fig 1d, iii). Next, the *BAC-Sprr2f-Cre* allele was introduced by crossing a *Chd4*^*fl/fl*^ dam with a sire hemizygous for the *BAC-Sprr2f-Cre* (Fig 1d, iv). Lastly, the resultant *BAC-Sprr2f-Cre*^*0/*^^+^*; Chd4*^*fl/*^^+^ male offspring were crossed with *Chd4*^*fl-*^ homozygotes (Fig 1d, v) to produce *BAC-Sprr2f-Cre; Chd4*^*fl/fl*^ females (Fig 1d, vi). The resultant *BAC-Sprr2f*^*0/*^^+^ *Chd4*^*fl/fl*^ female offspring were conditional knockout (cKO) mice, lacking CHD4 expression in the Cre-expressing, SPRR2F-positive cells of the endometrial epithelium. The presence of the *Chd4*^*fl-neo*^ (Fig 1e), *R26*^*Fki*^ (Fig 1f), *Chd4*^*fl*^ (Fig 1g), wild-type (Fig 1g), and *Cre* (Fig 1h) alleles was confirmed by PCR.

### *BAC-Sprr2f-Cre* is expressed exclusively in the endometrial epithelium and is absent in the oviducts and ovaries

**Fig 2:**
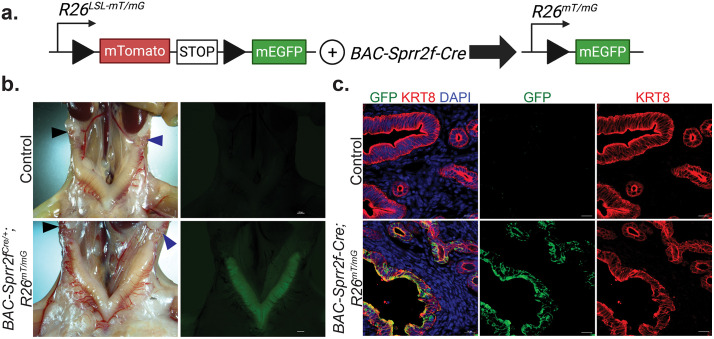
*BAC-Sprr2f-Cre* drives Cre-mediated recombination in the endometrial epithelium. **a.** Schematic of the *R26*^*mT/mG*^ allele. The R26^LSL-mT/mG^ allele consists of a membrane-targeted Tomato fluorescent reporter (mT) and a Lox-STOP-Lox (LSL) cassette, flanked by lox P sites. Before Cre-recombination, all cells ubiquitously express mTomato fluorescence (channel not shown). In SPRR2F- expressing cells, BAC-Sprr2f-Cre-mediated recombination excises the LSL cassette, driving a switch from mTomato to membrane-targeted enhanced green fluorescent protein (mEGFP/mG). **b.** Photomicrograph of uteri from 12-week-old control (*BAC-Sprr2f-Cre^0/+^; R26*^*+/+*^) and *BAC-Sprr2f-Cre^0/+^; R26*^*mT/mG*^ mice. Cre-positive cells within the endometrial epithelium fluoresce green in *BAC-Sprr2f- Cre^0/+^; R26*^*mT/mG*^ mice. Cre expression is absent in the oviducts and ovaries. Scale bar = 100 µm. **c.** Immunofluorescence staining for epithelial marker keratin 8 (KRT8-AF647 pseudo-colored red), green fluorescent protein (GFP), and DAPI nuclear stain was performed on uterine cryosections (from the mice in **b**). In the three-color merged image, co-expression is shown as yellow. The *BAC-Sprr2f-Cre* allele expression drives *R26*^*mT/mG*^ expression exclusively in the endometrial epithelium and is absent in the stroma. Maximum intensity projection images at 400x magnification; scale bar = 10 µm.

The *R26*^*mT/mG*^ fluorescent Cre-reporter allele (JAX #: 007676) was used to characterize the activity of *BAC-Sprr2f-Cre* across the female reproductive tract in 12-week-old *BAC-Sprr2f-*^*Cre^0^*^^*/+*^*; R26*^*mT/mG*^ and control (*BAC-Sprr2f-*^*Cre^0^*^^^*/+*^^*; R26*^*+/+*^) mice [30]. In mice with the *R26*^*LSL-mT/mG*^ allele, membrane-targeted fluorescent Tomato is ubiquitously expressed in all cells. In *BAC-Sprr2f-*^*Cre^0^*^^^*/+*^^*; R26*^*mT/mG*^ mice, Cre-recombination occurs in SPRR2F-expressing cells, resulting in a switch from mTomato to enhanced green fluorescent protein (EGFP) expression (Fig 2a). Expression of the *BAC-Sprr2f-Cre* allele begins at the onset of puberty in a stochastic manner, with initial reports finding a 50% recombination in 6-week-old mice [26]. In concordance with previously published results, *BAC-Sprr2f-Cre* was expressed exclusively in the endometrial epithelium, and expression was absent in the endometrial stroma, oviducts, and ovaries (Fig 2b) [26]. The variegated expression characteristic of the *BAC-Sprr2f-Cre* allele appears stronger in the luminal epithelium compared to the glandular epithelium by anti-GFP IHC in *BAC-Sprr2f-*^*Cre^0^*^^^*/+*^^*; R26*^*mT/mG*^*; Arid1a*^*fl/fl*^ mice when compared to control mice (Fig 2c).

### *BAC-Sprr2f-Cre* drives variegated loss of CHD4 exclusively in the endometrial epithelium

**Fig 3:**
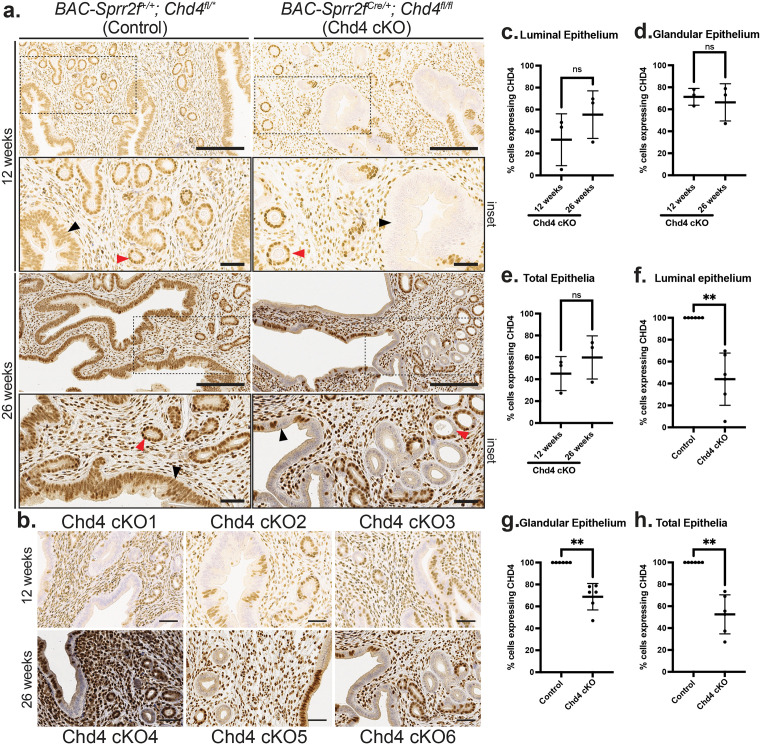
*BAC-Sprr2f-Cre* induces variegated knockout of *Chd4* in the endometrial epithelium. **a.** CHD4 loss was assessed by anti-CHD4 immunohistochemistry (IHC). Representative images of the endometria from 12- and 26-week-old *Chd4* cKO (*BAC-Sprr2f-Cre^0/+^; Chd4*^*fl/fl*^) and (*BAC-Sprr2f-Cre*^*+/+*^*; Chd4*^*fl/fl*^ or *Chd4*^*fl/+*^) control mice. Images were acquired at 20x magnification; Scale bar = 200 µm. Dashed boxes indicate regions shown at 40x magnification in the inset below each image (scale bar = 50 µm). The black and red arrowheads label the luminal and glandular epithelia, respectively. **b.** Representative images of anti-CHD4 IHC showing the variation in CHD4 expression in the 12-week-old (cKO1–3) and 26-week-old (cKO4–6) mice. Images were taken at 40x magnification; scale bar = 50 µm. **c.**-**e.** The percentage of CHD4 expression between 12- and 26-week-old *Chd4* cKO mice was assessed in the luminal (**c**), glandular (**d**), and total (**e**) epithelia. **f.**-**h.** Comparison of the percentage of CHD4 expressing cells between *Chd4* cKO (n = 6) and control (n = 6) mice in the luminal (**f**), glandular (**g**), and total (**h**) epithelia. **c.**-**h.** The percentage of CHD4-expressing cells was calculated as: the number of CHD4-positive cells divided by the total number of cells multiplied by 100% and was calculated for the luminal, glandular, and total epithelial cells. In all scatterplots, the horizontal line represents the mean; error bars indicate ± 1 standard deviation. The statistic used was a two-tailed, unpaired t test. Welch’s Correction was only used for unequal variance. Significance thresholds: ns = not significant; p < 0.05 = *, p < 0.01 = **, p < 0.001 = ***, p < 0.0001 = ****.

We have created a novel mouse model of endometrial epithelial-specific CHD4 loss driven by *BAC-Sprr2f-Cre*. For the initial pilot cohort, three *Chd4* conditional knockout (cKO, *BAC-Sprr2f-Cre^0/+^; Chd4*^*fl/fl*^) mice and three Cre-negative control (*BAC-Sprr2f-Cre*^*+/+*^*; Chd4*^*fl/fl*^ or *Chd4*^*fl/+*^) mice were sacrificed at 12 weeks of age to confirm loss of CHD4 in endometrial epithelium and to assess endometrial histology. The 12-week-old mice from the pilot cohort were not estrous cycle staged. Knowing that the expression of the *BAC-Sprr2f-Cre* allele is estrogen-dependent and cumulative estrogen exposure increases with age, we wanted to examine the extent of CHD4 loss in a cohort of 26-week-old diestrus-staged *Chd4* cKO (n = 3) and control (n = 3) mice [26]. In both 12- and 26-week-old mice, control mice showed strong nuclear expression of CHD4 in the luminal and glandular epithelia (Fig 3a). The *Chd4* cKO mice at both ages exhibited variegated expression of CHD4 in the luminal and glandular epithelia, with notable variation observed between mice (Fig 3a-b). The extent of CHD4 loss was determined by manually counting DAB-positive and DAB-negative cells from anti-CHD4 IHC using Fiji/ImageJ. The percentage of cells expressing CHD4 was calculated as the number of CHD4 (DAB)-positive cells divided by the total number of cells (CHD4-positive + CHD4-negative) multiplied by 100% and was quantified for the luminal, glandular, and total (luminal + glandular) epithelia (Fig 3c-h). Therefore, the percentage of cells with CHD4 loss was calculated as 100% minus the percentage of cells expressing CHD4.

To assess whether age affects *BAC-Sprr2f-Cre* expression, we assessed the percentage of cells with and without CHD4 expression in 12- and 24-week-old *Chd4* cKO mice. At both 12 and 26 weeks old, *Chd4* cKO mice had a lower percentage of cells expressing CHD4, and therefore, a higher percentage of cells with CHD4 loss, in the luminal epithelium (Fig 3c) compared to the glandular epithelium (Fig 3d). There was no significant difference in the percentage of cells expressing CHD4 in the luminal (Fig 3c), glandular (Fig 3d), or total epithelia (Fig 3e) between 12- and 26-week-old *Chd4* cKO mice. With age-related differences in the extent of CHD4 loss ruled out, the 12- and 26-week-old mice were combined into six *Chd4* cKO and six control mice to assess the percentage of cells with CHD4 expression loss. The percentage of cells with CHD4 loss in the luminal (Fig 3f), glandular (Fig 3g), and total epithelia (Fig 3h) was 56.1.0%, 31.2%, and 47.5%, respectively, when compared to control mice (Table 2).

**Table 2:** Mean percentage of endometrial epithelial cells with and without CHD4 protein expression.

Epithelium	Genotype	% cells expressing CHD4	% cells with CHD4 Loss	p value
Luminal	Control	100.0	0.0	0.0022
	*Chd4* cKO	44.0	56.1	
Glandular	Control	100.0	0.0	0.0014
	*Chd4* cKO	68.8	31.2	
Total	Control	100.0	0.0	0.0013
	*Chd4* cKO	52.5	47.5	

### *Chd4* cKO mice exhibit normal endometrial architecture and histology

**Fig 4:**
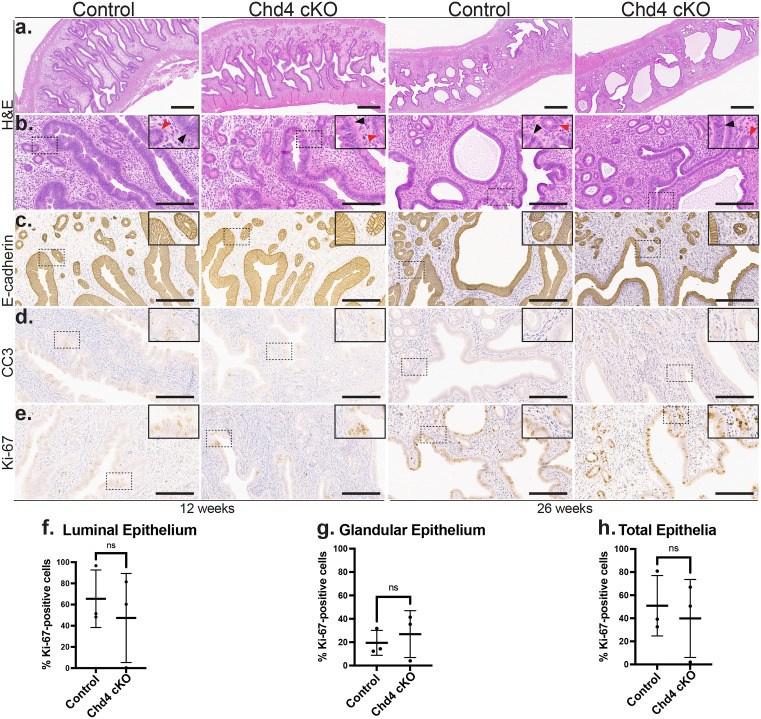
Chd4 cKO mice showed normal endometrial histology. **a.**-**b.** Representative images of hematoxylin and eosin (H&E) stained endometria from 12 and 26-week-old *Chd4* cKO and control mice. **a.** Low-power (4x magnification; scale bar = 600 µm) images showing similar endometrial architecture between genotypes at both 12- and 26-week-old mice. **b.** Higher magnification (20x magnification; scale bar = 200 µm) image of the slide shown in (**a**). D**a**shed boxes indicate regions shown at 40x magnification in the inset below each image. The black and red arrowheads label the luminal and glandular epithelia, respectively. **c.**-**e.** Representative images of IHC staining of endometria from 12- and 26-week-old *Chd4* cKO and control mice. Images were acquired at 20x magnification; Scale bar = 200 µm. IHC was performed to assess the expression of epithelial (E-cadherin) (**c**), apoptotic (Cleaved Caspase 3/CC3) (**d**), and proliferative (Ki-67) (**e**) markers between genotypes at 12 and 26 weeks of age. **f.**-**h.** The percentage of Ki-67 expression was assessed in the luminal (**f**), glandular (**g**), and total (**h**) epithelia of the diestrus-staged, 26-week-old mice (n = 3 *Chd4* cKO; n = 3 control). The percentage of cells expressing Ki-67 was calculated as the number of Ki-67-positive cells divided by the total number of cells multiplied by 100% and was calculated separately for the luminal, glandular, and total epithelia. In all scatterplots, the horizontal line represents the mean; error bars indicate ± 1 standard deviation. The statistic used was a two-tailed, unpaired t test. Significance thresholds: ns = not significant; p < 0.05 = *, p < 0.01 = **, p < 0.001 = ***, p < 0.0001 = ****.

Hematoxylin and eosin (H&E) staining showed no apparent differences in the endometrial architecture between *Chd4* cKO and control mice at 12 or 26 weeks of age (Fig 4a). *Chd4* cKO mice had normal endometrial histology by H&E staining, similar to that of control mice at both 12 and 26 weeks of age (Fig 4b). The expression of epithelial marker E-cadherin appeared similar in the glandular and luminal epithelia between genotypes in the 12- and 26-week-old mice (Fig 4c). IHC showed minimal expression of cleaved caspase 3 (CC3) in both 12- and 26-week-old mice, independent of genotype (Fig 4d). (Fig 4c-d). Variable expression of Ki-67 occurred across the non-estrous cycle staged 12-week-old mice from the pilot cohort, consistent with known changes in proliferation throughout the murine estrous cycle(Fig 4e) [39]. The expression of Ki-67 was assessed in the diestrus-staged 26-week-old *Chd4* cKO and control mice (Fig 4e). There was no significant difference in the percentage of Ki-67-positive cells in the luminal (Fig 4f), glandular (Fig 4g), or total (Fig 4h) epithelia between 26-week-old *Chd4* cKO and control mice.

### *Chd4* cKO mice are fertile and have no difference in fertility when compared to control mice

**Fig 5:**
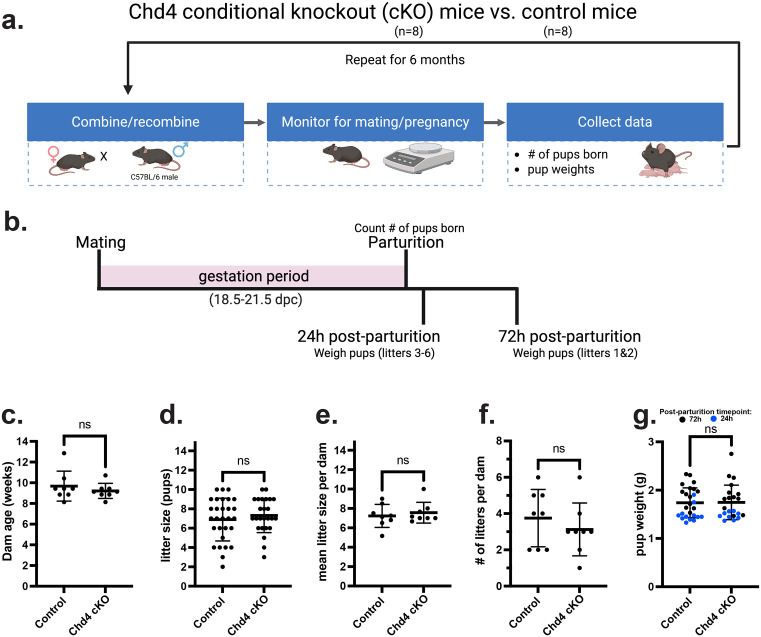
*Chd4* conditional knockout mice show no difference in fertility compared to control mice. **a.** Visual summary of experimental design for the *Chd4* breeding trial. **b.** Representative timeline of mouse pregnancy containing both timepoints to assess pup weight: 24 and 72 hours post-parturition. **c.** There was no difference in dam age between genotypes upon starting the breeding trial. **d.** There was no difference in the average litter size between genotypes. Each datapoint represents one litter. **e.** There was no difference in the average litter size per dam between genotypes. Each datapoint represents one dam. **f.** There was no genotype-specific difference in the number of litters per dam. **g.** There was no difference between genotypes in pup weight at 24 or 72 hours post-parturition or when the timepoints were combined. Blue and black datapoints denote pups weighed 24 and 72 hours post-parturition, respectively. **c.**-**g.** In all scatterplots, the horizontal line represents the mean; error bars indicate ± 1 standard deviation. The statistic used was a two-tailed, unpaired t-test. Significance thresholds: ns = not significant; p < 0.05 = *, p < 0.01 = **, p < 0.001 = ***, p < 0.0001 = ****.

The functional effects of CHD4 loss on endometrial function were assessed by a 6-month breeding trial in which *Chd4* cKO (n = 8) and control (n = 8) dams were serially bred to a C57BL/6 sire (Fig 5a). Over the course of the breeding trial, all *Chd4* cKO and control dams successfully mated and gave birth to live pups, which were weighed 24 or 72 hours post-parturition (Fig 5b). Dam age ranged from 8 to 14 weeks of age at the start of the trial, and there was no significant difference in the dam age between genotypes (Fig 5c). There was no difference in the average litter size (Fig 5d), the average litter size per dam (Fig 5e), the number of litters per dam (Fig 5f), or mean pup weight between genotypes when weighed 24 or 72 hours post-parturition or when combined (Fig 5g)(Table 3).

**Table 3:** Breeding trial summary.

Fertility Metric	Control	*Chd4* cKO	p value
Dam age at start of trial	9.679(±1.442)	9.214(±0.7324)	0.4305
Litter size (pups)	6.897 (±2.209)	7.320 (±1.773)	0.4458
mean litter size per dam	7.230 (±1.184)	7.563 (±1.073)	0.5656
number of litters per dam	3.750 (±1.581)	3.125 (±1.458)	0.4249
Pup weight (g)	1.738 (±0.310)	1.747 (±0.358)	0.9266

## Discussion

Somatic mutations in *CHD4* are found in the endometria of healthy women and those with endometrial cancer [7,40]. Knowing that endometrial carcinomas and other pathologies of the endometrium, including endometrial hyperplasia [6] and endometriosis [41], are thought to arise from the endometrial epithelium, we created an endometrial epithelial-specific mouse model of conditional CHD4 loss. Through targeted breeding, FLP1 recombination excised the FRT site-flanked *lacZ* reporter and neomycin cassette, creating the *Chd4*^*fl*^ allele. At the onset of puberty, *BAC-Sprr2f-Cre* induced Cre recombination of the *Chd4*^*fl*^ allele, leading to conditional loss of CHD4 in the Cre-expressing cells of the endometrial epithelium. Mice with conditional *Chd4* knockout exhibited a variegated expression pattern characteristic of *BAC-Sprr2f-Cre* activity reported in the literature [26,42]. Although variegated, CHD4 loss was more extensive in the luminal epithelium than in the glandular epithelium and did not change with age. *Chd4* cKO mice did not show any gross anatomical or histological differences when compared to control mice. Endometrial function was assessed by a 6-month breeding trial, which showed no difference in fertility between genotypes. Collectively, these results demonstrate that variegated CHD4 loss does not alter endometrial structure or function in 6-month-old mice. Is this lack of phenotype due to the incomplete knockout caused by variegation, or is *Chd4* dispensable for endometrial epithelial function?

In mice, germline knockout of *Chd4* (*Chd4*^*-/-*^) is embryonic lethal due to implantation failure [43]. In contrast, *Chd4*^*+/-*^ (heterozygous) mice survive into adulthood but show altered growth, as well as altered neurological, cardiovascular, and reproductive development [44], similar to what is observed in Sifrim-Hitz-Weiss syndrome in humans [45,46]. Importantly, mice heterozygous for CHD4 loss can still reproduce, which may explain why the *Chd4* cKO mice with 52.5% remaining CHD4 expression in endometrial epithelia exhibited normal fertility. While CHD4 expression is haploinsufficient during development, the consequences of post-developmental *CHD4* loss appear to be tissue specific. In malignancy, *CHD4* has context-dependent functions as a tumor suppressor and an oncogene. *CHD4* overexpression is associated with oncogenic activity, poor prognosis, and increased risk of colorectal cancer and ovarian cancer metastases [23,25]. In endometrial carcinoma, *CHD4* has been reported to have both tumor suppressive [20] and oncogenic activity [47], supporting the notion that *CHD4* gene dosage may be key to understanding the context-dependent CHD4 activity.

*BAC-Sprr2f-Cre* was selected due to its reported specificity to the endometrial epithelium and absence in the endometrial stroma, myometrium, ovaries, oviducts, and kidneys [26]. To elucidate the extent to which variegated CHD4 loss contributed to the observed absence of a phenotype, an alternative endometrial epithelial-specific Cre driver, such as Lactoferrin i-Cre (*Ltf-iCre*), could be used to further evaluate the extent to which endometrial epithelial CHD4 is required for endometrial epithelial structure and function [48]. However, lactoferrin is expressed in other tissues, including mammary glands and neutrophils, the off-target effects of which may alter endometrial functions such as fertility [48,49].

The majority of CHD4 mutations in endometrial carcinoma are missense mutations and are thought to lead to reduced or loss of CHD4 function [20,21,50]. By targeting *Chd4* knockout to the endometrial epithelium, we took a functional approach to both model the purported effects of these mutations and understand the tissue-specific consequences of CHD4 loss in this cell type. Phenotypic differences between somatic mutation and deletion of cancer-associated genes have been reported, including differences in tumorigenicity between *TP53*^*R172H*^ mutation and *TP53*^*fl*^ in a mouse model of *KRAS*^*G12V*^-mutant rhabdomyosarcoma [51]. Accordingly, it is conceivable that the consequences of a *CHD4* mutation, even a loss-of-function mutation, may not be entirely congruent with the effects of a conditional knockout, as modeled here.

Endometrial carcinoma most commonly arises in peri- and post-menopausal women [50]. The mice used in this study were still cycling and, therefore, may not have represented the atrophic endometrial epithelium common in post-menopausal women. Further, CHD4 is always co-mutated with other cancer driver genes and is frequently co-mutated with *PTEN*, *PIK3CA*, *PIK3R1*, *ARID1A*, *TP53*, and *KRAS* [50]. Many chromatin remodeling gene mutations are necessary but not sufficient to drive endometrial tumorigenesis, suggesting that *CHD4* may require an additional genetic alteration or activating mutation to promote tumor formation [11,52,53].

## Supporting information

S1_raw_images Fig 1e-f raw images. Raw images of the genotyping gels shown in Fig 1e-f. Primer sequences, product sizes, annealing temperatures, as well as genotyping protocol source, are also shown.
(PDF)

S1 Data Minimal Dataset. Raw data used to generate graphs shown in Figs 3, 4, and 5.
(XLSX)
