# Supplementary material for: Generation and characterization of a mouse model of conditional Chd4 knockout in the endometrial epithelium
Source: PLoS One. 2025 Dec 19;20(12):e0326723. doi: 10.1371/journal.pone.0326723 (PMC12716746; doi:10.1371/journal.pone.0326723)
Supplement: S1_raw_images — Primer sequences, product sizes, annealing temperatures, as well as genotyping protocol source, are also shown. (PDF) [file pone.0326723.s001.pdf]

Figure 1e.	Lane	Sample	Product size
	1	Ladder	100-1000 bp
	2	water control	no band
	3	Chd4$^{+/+}$	no band
	4	*Chd4*$^{fl\text{-}neo/+}$	536 bp

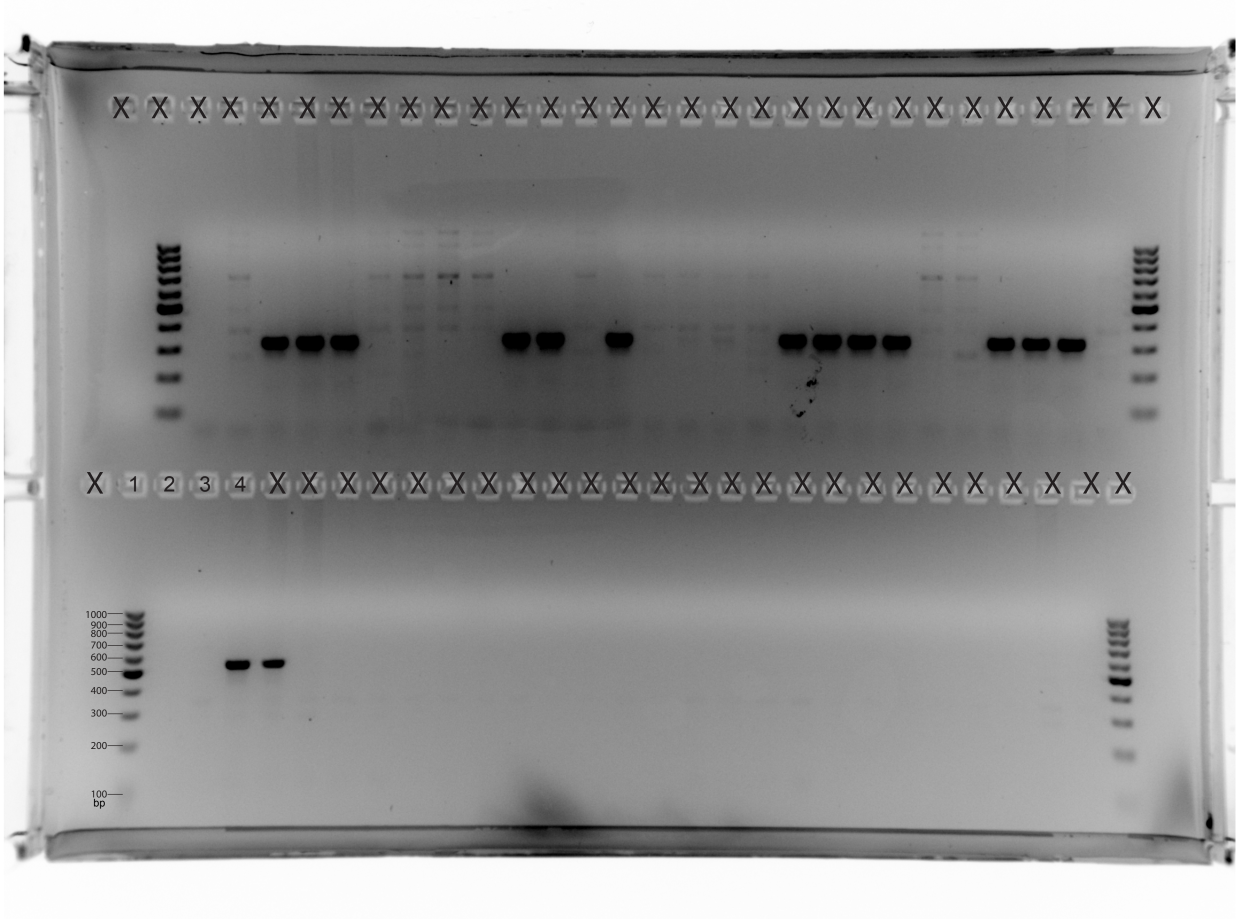

Genotyping Target	Primer Name	Sequence	Product size	Annealing temperature	Source
tm1a (*Chd4*$^{fl\text{-}neo}$)	2. 37690-neoF	GGGATCTCATGCTGGAGTTCTTCG	536 bp	58°C	(1)
	4. 37690-TTR	ACCTGCAGCCTACTGCCATGG			

	Lane	Sample	Product size
Figure 1f.	1	Ladder	100-1000 bp
	2	*R26*$^{Fki/+}$	725 bp
	3	*R26*$^{+/+}$	No band

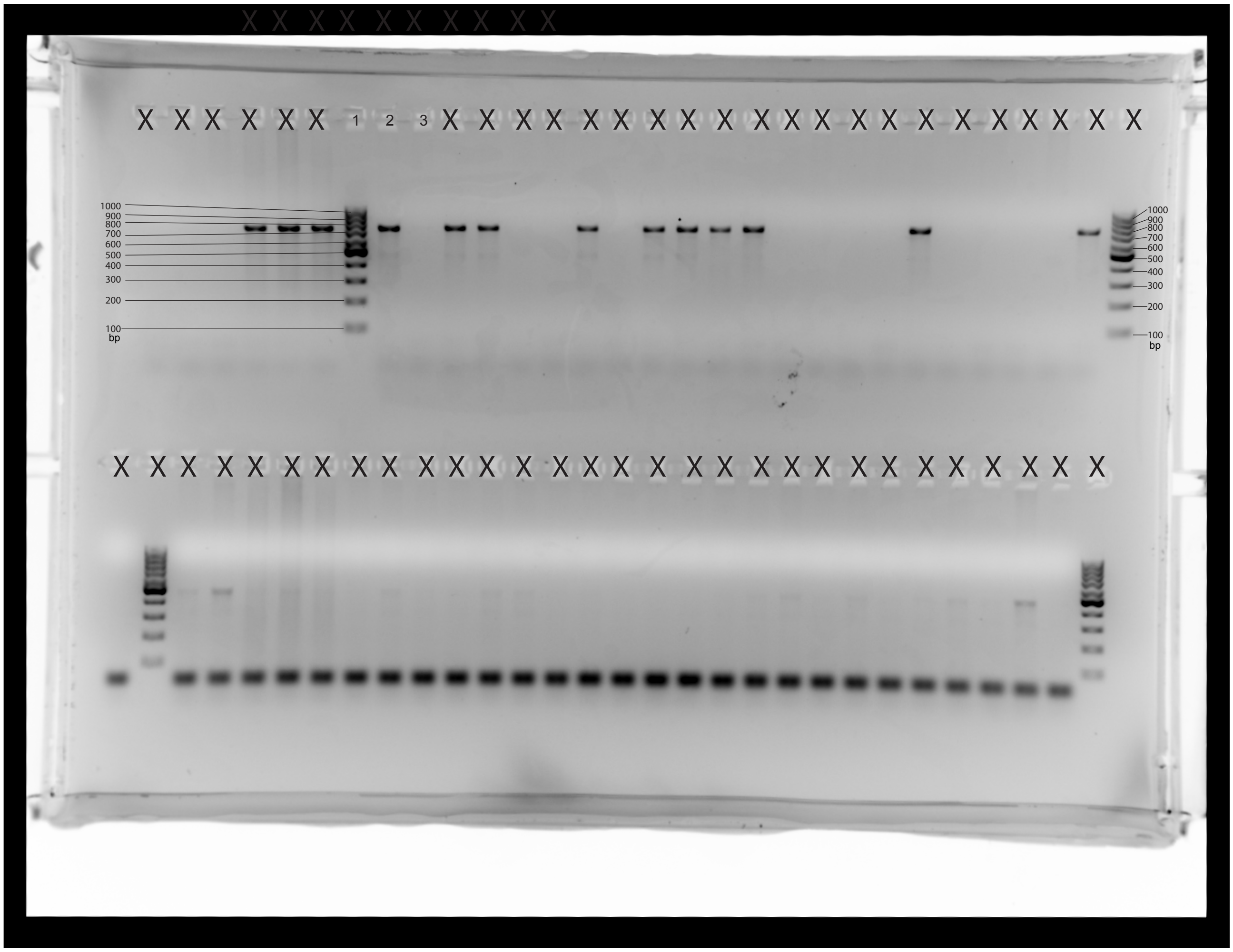

Genotyping Target	Primer Name	Sequence	Product size	Annealing temperature	Source
R26^{Fki} (mutant allele)	oIMR1348-MutantF	CAC TGA TAT TGT AAG TAG TTT GC	725 bp	58°C	(2)
	oIMR1349-MutantR:	CTA GTG CGA AGT AGT GAT CAG G			

	Lane	Sample	Product size(s)
Figure 1g.	1	Ladder	100-1000 bp
	2	*Chd4* $^{fl\text{-}only/fl\text{-}only}$	413 bp
	3	*Chd4* $^{fl\text{-}only/+}$	413 bp / 291 bp

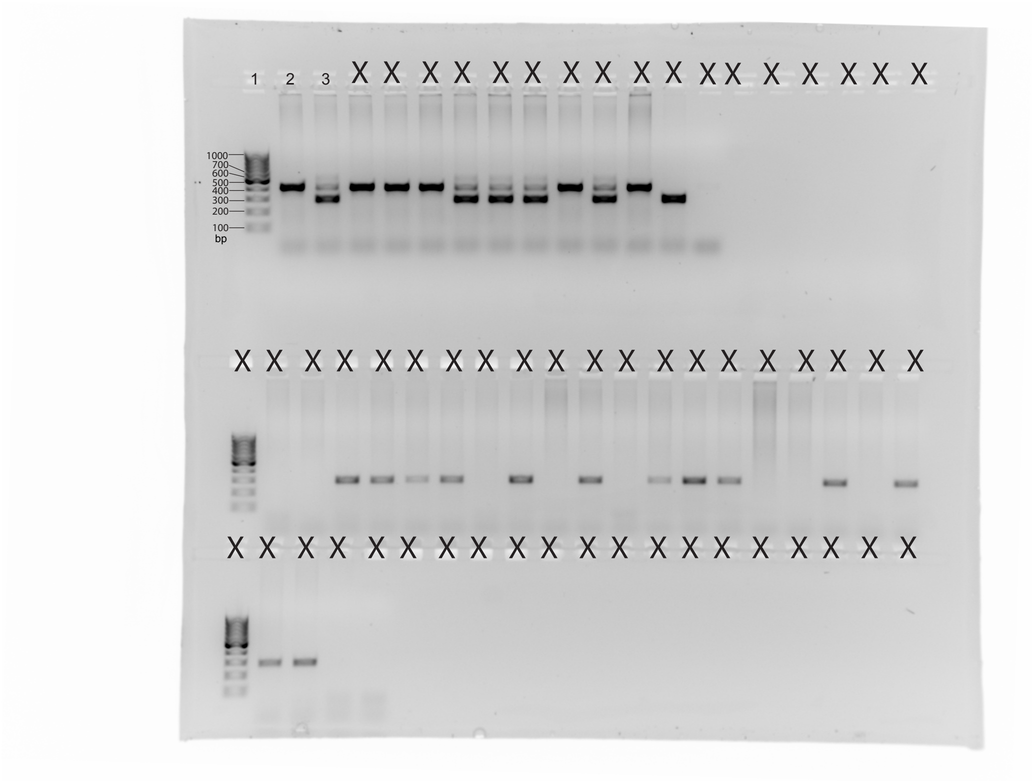

Genotyping Target	Primer Name	Sequence	Product size(s)	Annealing temperature	Source
tm1c (*Chd4* $^{fl\text{-}only}$)	6. 37690-F	GCAGTTCTGAGTGTAAGGTCAGTCTGG	413 bp	58°C	(1)
Wild-type (*Chd4* $^{+}$)	4. 37690-TTR	ACCTGCAGCCTACTGCCATGG	291 bp		

	Lane	Sample	Product size
Fig 1h.	1	Ladder	100-1000 bp
	2	*Cre/+*	~300 bp
	3	+/+	no band

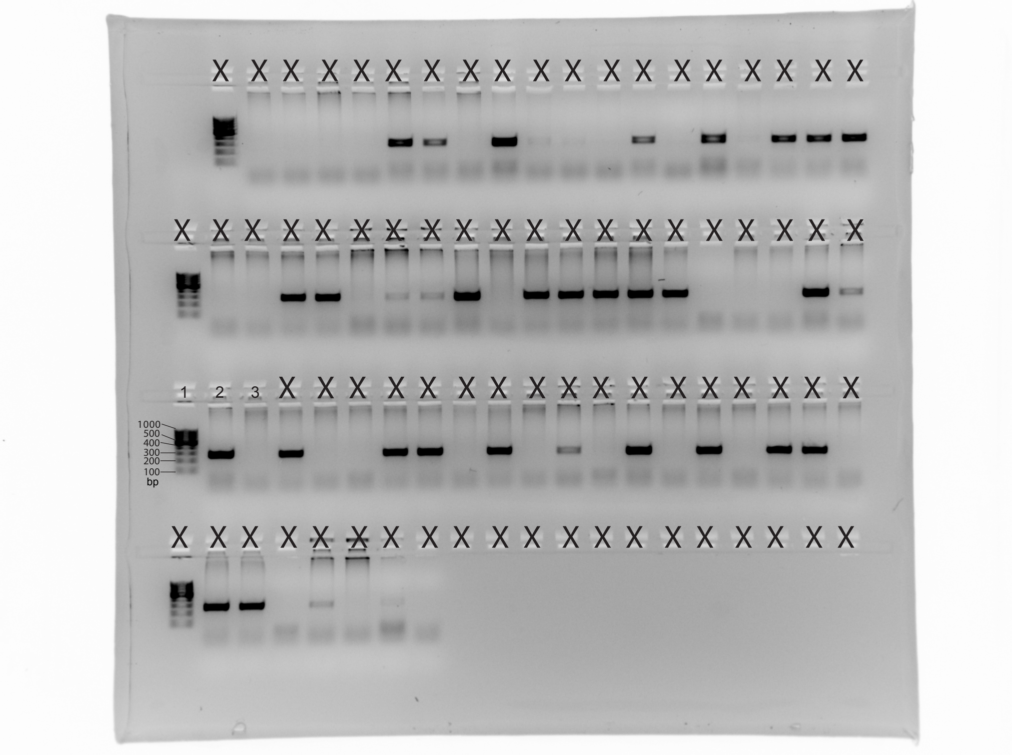

Genotyping Target	Primer Name	Sequence	Product size	Annealing temperature	Source
Cre	1019759753 Cre-F2	TGCAACGAGTGATGAGGTTC	~300 bp	55°C	(3)
(transgene)	1019759754 Cre-R2	ACAGCATTGCTGTCACTTGG			

References:

1. (MMRRC) MMRRC. Genotyping protocol for Stock No. 037690: University of California, Davis; n.d. [Available from: https://mmrrc.ucdavis.edu/protocols/037690Geno_Protocol.pdf.
2. Laboratory TJ. Genotyping Protocol 26666: Separated PCR Assay - Gt(ROSA)26Sor<tm1(FLP1)Dym> Version 2.2 2025 [Available from: https://www.jax.org/Protocol?stockNumber=016226&protocolID=26666.
3. Shpargel KB, Starmer J, Yee D, Pohlers M, Magnuson T. KDM6 demethylase independent loss of histone H3 lysine 27 trimethylation during early embryonic development. PLoS Genet. 2014;10(8):e1004507.
